# Video-assisted functional assessment of index pollicisation in congenital anomalies

**DOI:** 10.1007/s11832-016-0756-2

**Published:** 2016-06-28

**Authors:** Virginie Mas, Brice Ilharreborde, Cindy Mallet, Keyvan Mazda, Anne-Laure Simon, Pascal Jehanno

**Affiliations:** Paediatric Orthopaedic Department, Robert Debré Hospital, Assistance Publique des Hôpitaux de Paris (AP-HP), Paris Diderot University, 48 Bd. Sérurier, 75019 Paris, France

**Keywords:** Functional assessment, Index pollicisation, Thumb hypoplasia/aplasia, Video assistance

## Abstract

**Purpose:**

Functional results of index pollicisation are usually assessed by the clinical score of Percival. This score is based on elementary hand movements and does not reflect the function of the neo thumb in daily life activities. The aim of this study was to develop a new video-assisted scoring system based on daily life activities to assess index pollicisation functional outcomes.

**Methods:**

Twenty-two consecutive children, operated between 1998 and 2012, were examined with a mean of 77 months after surgery. The mean age at surgery was 34 months. Post-operative results were evaluated by a new video-assisted 14-point scoring system consisting of seven basic tasks that are frequently used in daily activities. The series of tasks was performed both on the request of the examiner and in real-life conditions with the use of a hidden camera. Each video recording was examined by three different examiners. Each examiner rated the video recordings three times, with an interval of one week between examinations. Inter- and intra-observer agreements were calculated.

**Results:**

Inter- and intra-observer agreements were excellent both on request (κ = 0.87 [0.84–0.97] for inter-observer agreement and 0.92 [0.82–0.98] for intra-observer agreement) and on hidden camera (κ = 0.83 [0.78–0.91] for inter-observer agreement and 0.89 [0.83–0.96] for intra-observer agreement). The results were significantly better on request than on hidden camera (*p* = 0.045). The correlation between the video-assisted scoring system and the Percival score was poor.

**Conclusion:**

The video-assisted scoring system is a reliable tool to assess index pollicisation functional outcomes. The scoring system on hidden camera is more representative of the neo thumb use in daily life complex movements.

**Level of evidence:**

Level IV.

## Introduction

Pollicisation is a challenging surgical technique, which consists in the transposition of the index into the thumb position. This procedure is used for thumb congenital hypoplasia or aplasia reconstruction, and aims to create a neo pinch in order to restore opposition and improve daily life activities function [1–4]. Since the first description by Buck-Gramcko in 1971, several techniques have been developed, in order to optimise the neo thumb function [4–6]. Outcomes are considered satisfactory regarding range of motion, strength, cosmetic aspect and sensitivity of the neo thumb [1–4, 6]. However, post-operative functional assessment remains heterogeneous and not standardised, without clear guidelines [7–11] . Currently, the neo pinch is only assessed by static measurements and elementary movements of the Percival score, which might not reflect the actual function of the new thumb in daily activities [11]. In the management of patients with cerebral palsy, video-assisted scoring methods have progressively gained popularity in the analysis of both lower and upper limbs [12–14]. Inspired by these techniques, we developed an original video-assisted scoring system based on daily life activities to evaluate index pollicisation functional outcomes. The aim of this study was to assess the intra- and inter-observer agreement of this novel scoring system and to compare it to the Percival score.

## Materials and methods

### Patients

After institutional review board approval, 22 patients operated for index pollicisation between 1998 and 2012 were included. According to Blauth’s classification, indications were type IV hypoplasia in 12 cases, type IIIb hypoplasia in two cases and type V aplasia in eight cases [6]. The deformity was either isolated or associated with various anomalies (Table 1). The malformation was associated to other upper-limb anomalies in 59 % of the cases. The surgical procedure, as described by Buck-Gramcko, was performed by two experienced paediatric orthopaedic surgeons [5, 15]. Surgical treatment of wrist misalignment was previously performed for all the patients with an associated radial longitudinal deficiency. The average age at surgery was 34 ± 8 months (range 12–192). The mean follow-up was 77 ± 7 months (range 24–157). All data were collected after the parents signed an informed consent.

**Table 1:** Patients’ characteristics

ID	Gender	Age at OP (months)	Follow-up (months)	Diagnosis (Blauth)/associated anomalies	Side
1	Boy	12	120	Hypoplasia type IV isolated	R
2	Boy	22	85	Hypoplasia type V/Pierre Robin syndrome/radial club hand	L
3	Girl	22	92	Hypoplasia type IV isolated	R
4	Boy	52	98	Aplasia isolated	L
5	Boy	16	116	Aplasia isolated	R
6	Girl	18	76	Hypoplasia type IV isolated	R
7	Girl	20	84	Aplasia/radial club hand	L
8	Boy	31	32	Hypoplasia type IV isolated	R
9	Boy	20	65	Aplasia/radial club hand	R
10	Girl	16	62	Hypoplasia type IV/radial club hand	R
11	Girl	56	98	Hypoplasia type IV/Holt–Oram syndrome/radial club hand	L
12	Girl	25	62	Hypoplasia type IV isolated	R
13	Boy	18	78	Aplasia isolated	L
14	Girl	34	28	Aplasia/radial club hand	R
15	Girl	39	157	Hypoplasia type IV/Holt–Oram syndrome	R
16	Boy	14	118	Hypoplasia type IIIb isolated	L
17	Girl	45	83	Aplasia/radial club hand	R
18	Girl	16	87	Hypoplasia type IV/Fanconi syndrome	R
19	Boy	192	33	Hypoplasia type IV isolated	R
20	Girl	18	34	Hypoplasia type IV isolated	L
21	Boy	43	52	Hypoplasia type IV/Pierre Robin syndrome	R
22	Boy	19	24	Aplasia/radial club hand	L

### Video-assisted scoring system

The video-assisted evaluation consisted of seven basic tasks that are frequently used in daily activities: writing, drinking, eating, combing hair, moving an object from one point to another, moving an object from one hand to the other and, finally, one complex gesture (opening a bottle, tying shoelaces, dressing, doing up a button) to assess two-hand coordination. The patients were filmed in two different conditions: (a) on request of the examiner and (b) in real-life conditions with the use of a hidden camera. During the test in real-life conditions, the child was sitting in a playing room and ignored that he was being filmed. He/she was allocated to different stands by his/her parents. On the first table, various colouring books with markers were disposed for the assessment of writing. On the second table, the parents role played a dinner party to assess drinking and eating and moving objects. Finally, on the third table, dressing and styling accessories were arranged for the evaluation of the remaining activities. The surgeon stood behind and filmed the hand movements using a hidden camera. Each condition (on request and on hidden camera) took 15–30 min.

**Fig. 1:**
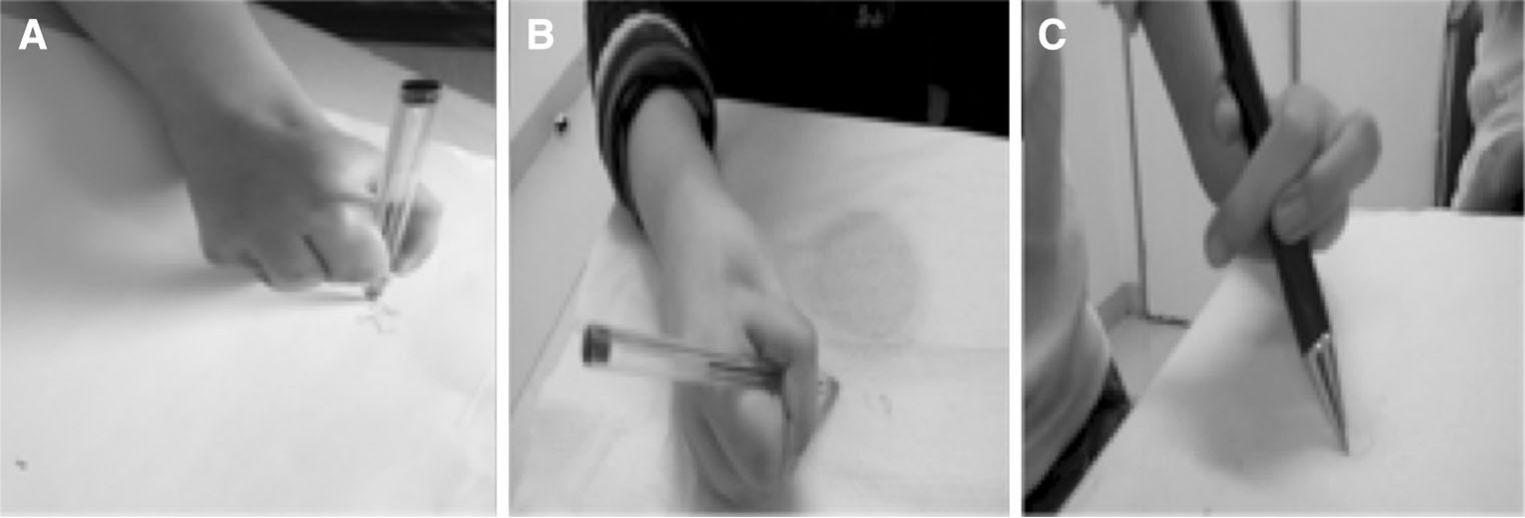
Illustration of the scoring system in three patients filmed while writing. Patient** a** was scored 2 points because he used his neo thumb and the pinch normally, patient** b** was scored 1 point because he used a lateral pinch of the neo thumb and patient** c** was scored 0 points because he did not use the neo thumb

Each video recording was analysed by three different examiners: an orthopaedic surgeon, who did not perform the surgery, a paediatrician and a physiotherapist. Each examiner analysed the video recordings three times, with an interval of one week between examinations. Video recordings were presented to the examiners in a random order. Each activity was scored 0–2 points (0: no use of the neo thumb, 1: partial use of the neo thumb and 2: normal use of the neo thumb with a pinch grip), leading to a total score of 14 points (Table 2; Fig. 1). The total score was defined as excellent (>11 points), good (8–11 points), fair (4–7 points) or poor (≤3 points).

**Table 2:** Video-assisted scoring system

Activities	
1. Writing	= 0-1-2
2. Drinking	= 0-1-2
3. Eating	= 0-1-2
4. Combing hair	= 0-1-2
5. Moving an object from one point to other	= 0-1-2
6. Moving an object from one hand to the other	= 0-1-2
7. Complex gesture (opening a bottle, tying shoelaces, dressing, doing up a button)	= 0-1-2
Total	/14 points

The test was recorded in two different conditions: on request of the examiner and in real-life conditions with the use of a hidden camera. Each activity was scored from 0 to 2 points (0: no use of the neo thumb, 1: partial use of the neo thumb and 2: normal use of the neo thumb with a pinch grip), leading to a total score of 14 points in each condition

### The Percival score

All patients were assessed with the traditional Percival score [11]. This score evaluates thumb function (tip pinch, pulp pinch, grasp, opposition and mobility), sensitivity and cosmetics [11]. Seven parameters are analysed on a 22-point score. The score was defined as excellent ≥20, good [16–19], fair [12–15] and poor <12 points (Table 3).

**Table 3:** The Percival score

Tip pinch (4 points)		
Strength (compared with normal)	<25 %	= 0
	25–75 %	= 1
	>75 %	= 2
Accuracy (pick up pin)	Unable	= 0
	With difficulty	= 1
	With ease	= 2
Pulp pinch (2 points)		
Strength (compared with normal)	<75 %	= 0
	>75 %	= 1
Accuracy (pick up key)		= 1
Opposition (3 points)		
To middle		= 1
To ring		= 1
To little		= 1
Grasp (3 points)		
Ability to grasp tennis ball		= 1
Ability to grasp table-tennis ball		= 1
Strength >75 %		= 1
Mobility (3 points)		
Active C.M.C. joint motion		= 1
Active M.P. joint motion		= 1
Active I.P. joint motion		= 1
Sensibility (3 points)		
Normal two-point discrimination		= 3
5–10 mm		= 2
>10 mm		= 1
Cosmetic (4 points)		
Length to within 0.5 cm of P.I.P. joint		= 1
Position (45°–80° abduction)		= 1
(90°–160° rotation)		= 1
Appearance considered good by parents		= 1

The total score was divided into excellent (>20 points), good (16–19 points), fair (12–15 points) and poor (≤12 points) results

### Statistical analysis

Inter- and intra-observer reliability of the video assistance scoring system was evaluated using a Kappa Cohen test. Excellent agreement and strong agreement were defined as κ-values between [0.81–1] and [0.61–0.8], respectively. The correlation between Percival score and the video-assisted scoring system was evaluated using the Spearman correlation test. Poor or no correlation was defined as *r*-values of [0–0.5] and 0, respectively. Fisher’s exact test or the χ^2^ test was used for the comparison of categorical variables. A *p*-value <0.05 was considered significant.

## Results

The inter- and intra-observer agreements were excellent both on request and on hidden camera conditions. On request condition, the kappa value was 0.87 [0.84–0.97] for inter-observer agreement and 0.92 [0.82–0.98] for intra-observer agreement. On hidden camera condition, the kappa value was 0.83 [0.78–0.91] for inter-observer agreement and 0.89 [0.83–0.96] for intra-observer agreement.

**Fig. 2:**
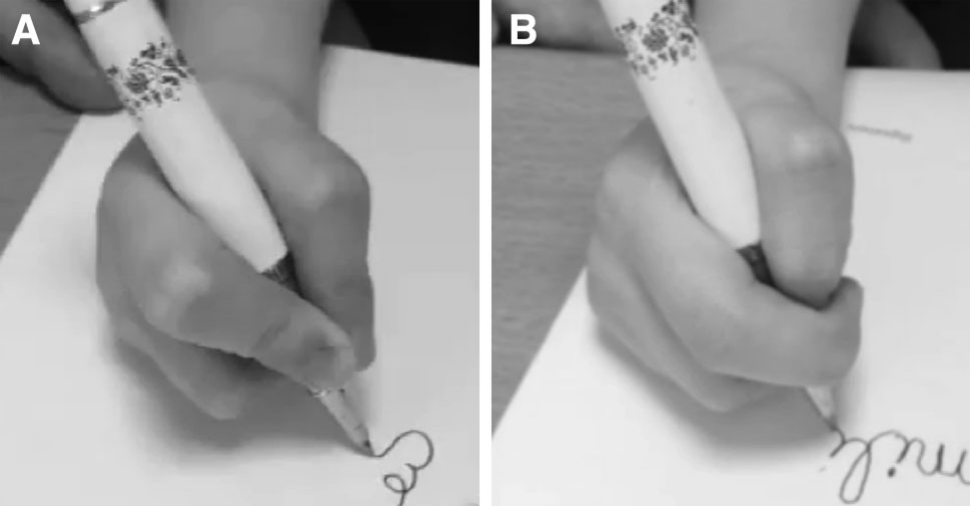
Patient filmed while writing on request (**a**) and on hidden camera (**b**). This patient used the pinch normally on request and a lateral pinch on hidden camera

The results were significantly better on request than on hidden camera. The score was good or excellent in ten patients on request condition compared with four on hidden camera condition with children who tended to use a lateral pinch (χ^2^ test, *p* = 0.045) (Fig. 2).

The mean post-operative Percival score was 14.9 ± 6 (range 11–19). The results were excellent, good, fair and poor in 2, 12, 5 and 3 patients, respectively.

**Fig. 3:**
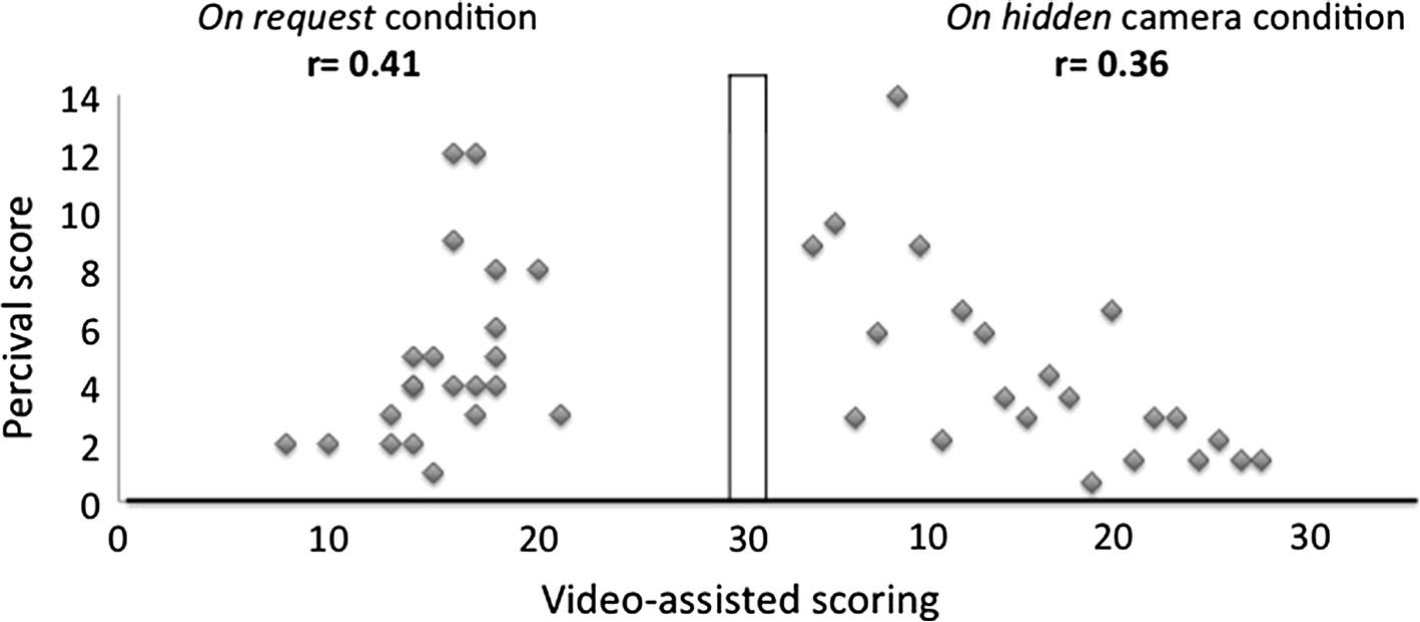
Correlation between the Percival score and the new video-assisted scoring, on request and on hidden camera conditions

The correlation between the Percival score and the video assistance scoring was poor: *r* = 0.41 when the video-assisted system was performed on request and *r* = 0.36 when it was performed on hidden camera (Fig. 3).

## Discussion

We developed a video-assisted scoring system to assess functional results in real-life activities of index pollicisation and showed that this test has excellent inter- and intra-observer agreement. We also showed that this video-assisted scoring system correlated poorly with the Percival score.

The essential aim of the pollicisation procedure is to restore an active pinch [16–18]. In our video-assisted scoring system, we selected seven activities representative of daily life activities to assess the results of pollicisation. These activities require the use of the thumb pinch. For example, the pulp pinch is used while ‘writing,’ the lateral pinch while ‘eating’ and the grip while ‘drinking.’ The realisation of complex gestures represents bimanual coordination. All these conditions are essential for a correct social integration [17–19]. These tasks were performed both on request and in real-life conditions using a hidden camera, to assess whether patients used their neo thumb in the same way in both conditions. We found that children tended to use their neo thumb correctly on request but tended to use inappropriately a lateral pinch on hidden camera condition. This explains why the results of pollicisation were better on request than on hidden camera. This also suggests that improvements in the surgical technique might improve the inappropriate use of the pinch [20, 21]. In this respect, pollicisation with tendon transfers using an abductor digiti minimi or a flexor digiti superficial of the fourth finger have been described to improve the neo thumb opposition [22, 23].

Results of the neo pinch are usually assessed by the Percival score [11]. In our study, the mean Percival score was similar to values reported in the literature, with 55 % excellent or good results [7, 9, 10, 16]. The poor correlation between our video-assisted scoring system and the Percival score may be explained by the fact that the Percival score evaluates range of motion for simple movements, strength, cosmetic aspect and sensitivity of the thumb [11], but does not reflect with enough relevance the accurate function of the neo thumb in daily activities. Therefore, post-operative results are often too optimistic in clinical reports when assessed by the Percival score.

### Limitations

The current study has several limitations. First, this video-assisted scoring system has not been validated in an independent population of patients, a necessary step before recommending its use in routine clinical practice. Second, unlike the Percival score, our score does not assess the cosmetic aspect of surgery. However, the cosmetic assessment of the scar and the length of the neo thumb could be introduced in a new version of the score. Third, the video-assisted scoring system is relatively time consuming (around 30 min).

In conclusion, our study introduces a new reliable video-assisted scoring system method for the assessment of index pollicisation functional outcomes and highlights the specific importance of the hidden camera analysis, which is more representative of the neo thumb use in daily life activities.

## References

[1] Gosset J, Sels M (1964). Technic, indications and results of pollicization of the 4th finger. Ann Chir.

[2] Tubiana R, Duparc J (1960). A new procedure for reconstruction of a sensitive thumb. Mem Acad Chir (Paris).

[3] Littler JW (1976). On making a thumb: one hundred years of surgical effort. J Hand Surg Am.

[4] Buck-Gramcko D (1971). Pollicization of the index finger. Method and results in aplasia and hypoplasia of the thumb. J Bone Joint Surg Am.

[5] Buck-Gramcko D (1977). Thumb reconstruction by digital transposition. Orthop Clin North Am.

[6] Blauth W (1967). The hypoplastic thumb. Arch Orthop Unfallchir.

[7] Sykes PJ, Chandraprakasam T, Percival NJ (1991). Pollicisation of the index finger in congenital anomalies. A retrospective analysis. J Hand Surg Br.

[8] Kozin SH (2012). Pollicization: the concept, technical details, and outcome. Clin Orthop Surg.

[9] Vekris MD, Beris AE, Lykissas MG, Soucacos PN (2011). Index finger pollicization in the treatment of congenitally deficient thumb. Ann Plast Surg.

[10] Ceulemans L, Degreef I, Debeer P, De Smet L (2009). Outcome of index finger pollicisation for the congenital absent or severely hypoplastic thumb. Acta Orthop Belg.

[11] Percival NJ, Sykes PJ, Chandraprakasam T (1991). A method of assessment of pollicisation. J Hand Surg Br.

[12] Davids JR, Peace LC, Wagner LV, Gidewall MA, Blackhurst DW, Roberson WM (2006). Validation of the Shriners Hospital for Children Upper Extremity Evaluation (SHUEE) for children with hemiplegic cerebral palsy. J Bone Joint Surg Am.

[13] Krumlinde-Sundholm L, Eliasson A-C (2003). Development of the Assisting Hand Assessment: a Rasch-built measure intended for children with unilateral upper limb impairments. Scand J Occup Ther.

[14] Viehweger E, Zürcher Pfund L, Hélix M, Rohon MA, Jacquemier M, Scavarda D, Jouve JL, Bollini G, Loundou A, Simeoni MC (2010). Influence of clinical and gait analysis experience on reliability of observational gait analysis (Edinburgh Gait Score Reliability). Ann Phys Rehabil Med.

[15] Flatt AE (1994). The care of congenital hand anomalies.

[16] Clark DI, Chell J, Davis TR (1998). Pollicisation of the index finger. A 27-year follow-up study. J Bone Joint Surg Br.

[17] James MA, Green HD, McCarroll HR, Manske PR (2004). The association of radial deficiency with thumb hypoplasia. J Bone Joint Surg Am.

[18] Riley SA, Burgess RC (2009). Thumb hypoplasia. J Hand Surg Am.

[19] Lamb DW (1977). Radial club hand. A continuing study of sixty-eight patients with one hundred and seventeen club hands. J Bone Joint Surg Am.

[20] Manske PR (2010). Index pollicization for thumb deficiency. Tech Hand Up Extrem Surg.

[21] Roper BA, Turnbull TJ (1986). Functional assessment after pollicisation. J Hand Surg Br.

[22] Kozin SH, Weiss AA, Webber JB, Betz RR, Clancy M, Steel HH (1992). Index finger pollicization for congenital aplasia or hypoplasia of the thumb. J Hand Surg Am.

[23] Loréa P, Medina J, Navarro R, Foucher G (2008). “Principalisation” of pollicization in congenital conditions. Technical modifications for functional and aesthetic improvement. Chir Main.

